# Paradigm shift required for translational research on the brain

**DOI:** 10.1038/s12276-024-01218-x

**Published:** 2024-05-01

**Authors:** Jong Hyuk Yoon, Dongha Lee, Chany Lee, Eunji Cho, Seulah Lee, Amaury Cazenave-Gassiot, Kipom Kim, Sehyun Chae, Edward A. Dennis, Pann-Ghill Suh

**Affiliations:** 1 Neurodegenerative Diseases Research Group, Korea Brain Research Institute, Daegu, 41062 Republic of Korea; 2 Cognitive Science Research Group, Korea Brain Research Institute, Daegu, 41062 Republic of Korea; 3 Department of Biochemistry and Precision Medicine Translational Research Program, Yong Loo Lin School of Medicine, National University of Singapore, Singapore, 119077 Singapore; 4 Singapore Lipidomics Incubator (SLING), Life Sciences Institute, National University of Singapore, Singapore, 117456 Singapore; 5 Research Strategy Office, Korea Brain Research Institute, Daegu, 41062 Republic of Korea; 6 Neurovascular Unit Research Group, Korean Brain Research Institute, Daegu, 41062 Republic of Korea; 7 Department of Pharmacology and Department of Chemistry and Biochemistry, University of California, San Diego, La Jolla, CA 92093-0601 USA; 8 Korea Brain Research Institute, Daegu, 41062 Republic of Korea

**Keywords:** Neuroscience, Medical research

## Abstract

Biomedical research on the brain has led to many discoveries and developments, such as understanding human consciousness and the mind and overcoming brain diseases. However, historical biomedical research on the brain has unique characteristics that differ from those of conventional biomedical research. For example, there are different scientific interpretations due to the high complexity of the brain and insufficient intercommunication between researchers of different disciplines owing to the limited conceptual and technical overlap of distinct backgrounds. Therefore, the development of biomedical research on the brain has been slower than that in other areas. Brain biomedical research has recently undergone a paradigm shift, and conducting patient-centered, large-scale brain biomedical research has become possible using emerging high-throughput analysis tools. Neuroimaging, multiomics, and artificial intelligence technology are the main drivers of this new approach, foreshadowing dramatic advances in translational research. In addition, emerging interdisciplinary cooperative studies provide insights into how unresolved questions in biomedicine can be addressed. This review presents the in-depth aspects of conventional biomedical research and discusses the future of biomedical research on the brain.

## Unlocking brain mysteries: high-throughput tools transform brain biomedical research

Biomedical research, which is the study of health and disease, has greatly improved our ability to manage diseases and has advanced global healthcare. However, understanding the causes of most brain disorders is still a challenge due to the limitations of traditional research methods. This study introduces a new translational research method that combines high-throughput analysis tools—which are techniques that can analyze a large amount of data quickly - like neuroimaging, multi-omics, and artificial intelligence (AI). The study suggests that combining neuroimaging, multi-omics, and AI can offer valuable insights, such as new disease markers, subtypes, and treatments. The authors believe this new method could overcome the limitations of traditional methods, leading to a better understanding of brain disorders and the development of new diagnostic tools and treatments.

This summary was initially drafted using artificial intelligence, then revised and fact-checked by the author.

## Introduction

Biomedical research has recently led to the successful management of several diseases, contributed to human health, and advanced the global healthcare agenda. Notably, biomedical research has successfully controlled diverse medical crises, including smallpox, pneumonia, and appendicitis^1^. Furthermore, mortality was reduced by approximately 48% from 1930 to the end of 1970, which is estimated to have resulted from active biomedical research^2^.

The field of biomedical research in the brain is still a budding research area with many unsolved questions^3^ first posed by ancient theoretical and religious concepts and now addressed by modern empirical research^4^. However, unlike diseases that have been solved with the advancement of modern science, the causes of most brain disorders remain unclear. This could be due to several specific limitations that are unique to conventional brain research, such as complex brain anatomy, difficulties obtaining human brain samples, the large gap between animal and human studies, and ethical limitations, especially in psychiatric disorders^5–7^. Approximately 100 years ago, physicist Emerson Pugh said, “If the human brain were so simple that we could understand it, we would be so simple that we could not.” Nevertheless, brain research has rapidly advanced over the past few decades in various fields, such as medical science, pharmacology, biology, and engineering^8^. Research in each discipline is maturing rapidly in terms of biomedical applications and development^9^. Notably, as the demand for high-performance tools has increased^10,11^, new high-performance materials^12,13^ and devices have been developed^14,15^. Moreover, cumulative databases have become invaluable references for all studies involving the identification of genomes, proteomes, and small molecules^16,17^. Recently, the integration and analysis of cumulative large-scale databases has become a tractable approach for understanding the brain connectome^18,19^.

The paradigm shift in translational research is a top-down method that preanalyzes patient-centered data and conducts data-based basic research and clinical/utilization research to overcome the limitations of conventional translational research. Collecting and analyzing patient-centered big data involves acquiring the maximum amount of data through various high-throughput analysis tools, enabling us to collect and merge data and derive meaningful results. Interdisciplinary collaboration leads to a comprehensive understanding^20^ and can provide new insights. Consequently, interdisciplinary collaborative research produces better results with synergistic benefits^21,22^. On this basis, interdisciplinary collaborative brain research can become a powerful framework for overcoming brain diseases through new approaches to translational research. Therefore, this review presents the in-depth aspects of conventional biomedical research and discusses the future of biomedical brain research.

## History of brain research

The interest in human brain research can be traced back to ancient times. The oldest known surgical text dates back to circa 1600 BCE in Egypt. It describes the restriction of head movement to treat trauma caused by a weapon, infection prevention methods, and bleeding control methods^23^. The first understanding of cranial structures, brain surface structures, and cerebrospinal fluid and 48 trauma cases were also recorded. Egyptians were believed to be knowledgeable about brain trauma and its importance. By the Renaissance, the anatomical diagram of the human brain was almost complete^24^. Since then, biomedical brain research has progressed to identifying brain structures, functions, and mechanisms. Therefore, empirical biomedical brain research has evolved according to time trends. In 1791, Luigi Galvani observed convulsions in frogs’ legs when they were in contact with a metal dissecting knife. This experiment was the first to show that an electric current can flow among living muscles, nerves, and cells and has become the basis for the development of electrophysiology^25^.

Camillo Golgi developed Golgi staining in the 1860s, and Ramon Cajal used it to reveal that neurons are the units that constitute the brain and are connected in a network. The discovery of neurons, the basic elements of the brain, in the early 20th century became the cornerstone of full-scale empirical brain research. Since then, many studies have investigated bioelectrical phenomena such as membrane potential, electrocardiography, electroencephalography, neuronal cell function, chemical transmission, nerve fibers, ion-transport mechanisms of nerve cell membranes, and cerebral function and information processing in the cerebral and visual cortex.

In the middle of the 20th century, empirical and detailed research was conducted on the behavior of neurons. This research elucidated the brain’s anatomical structure, but its information processing was poorly understood. It was later revealed that dozens of chemicals transmit information between neurons. In 1936, Henry Hallett Dale and Otto Loewi discovered and proved that acetylcholine was a chemical transporter of nerve impulses, and they won the Nobel Prize for their discovery^26^. In 1963, ion-based mechanisms associated with excitation and inhibition were discovered in the central and peripheral parts of the cell membrane, termed “action potentials” by John Carew Eccles, Alan Lloyd Hodgkin, and Andrew Fielding Huxley^27^.

The invention of the microscope led to the explosive development of biological research, including brain research. After Ernst Abbe’s diffraction limit (λ/2, where λ is the wavelength of light) was published in 1873, Stefan W. Hell developed stimulated emission depletion microscopy, which overcame the resolution limit of conventional optical microscopes using a laser excitation beam^28^. Since then, researchers have been able to observe objects <0.2 µm, enabling the understanding of viruses, interactions between individual proteins in cells, and even smaller molecules^29^. Since the early 2000s, superresolution fluorescence microscopy, especially stochastic optical reconstruction microscopy developed in 2006, has been used to randomly turn on and off fluorescent molecules to separate molecules on the time axis, overcoming the resolution limit and obtaining a high resolution of approximately 20 nm^30^. Furthermore, advances in molecular optics technology, including optical devices and sensors, have led to the development of nanoscopic techniques, enabling molecular and structural imaging of synapses and contributing to the study of physiological functions^31,32^. Since the brain is a large neural network, identifying the functions of each neuron is crucial. However, identifying the entire connected network and understanding its interactions are also critical^33^. Therefore, the working scale of research has moved from the microscale to the mesoscale^34^. Additionally, tissue transparency technology, which was first described in 2013, allows the internal structure of a given tissue to be observed in three dimensions after removing the lipid from the tissue and making it transparent, enabling observation of the entire brain^35^. Brain imaging at various scales has accelerated the study of neural circuits^36–38^. One of the most vital advancements in brain research has been the development of measurement equipment that can measure brain function externally and analyze the brain’s electrical signals. Since then, noninvasive brain imaging techniques such as in vivo and magnetic resonance imaging (MRI) have been developed. In 1973, Laterbur developed an imaging technique based on nuclear magnetic resonance technology; the technique was named nuclear magnetic resonance imaging and is now commonly referred to as MRI^39^. MRI enables noninvasive examination; provides information on the chemical structure of substances within a short examination duration; is useful for diagnosing diseases of the nervous system, such as the brain or spinal cord, which cannot be observed using radiographic scanning; and has great clinical value. The combination of MRI with positron emission tomography (PET), which displays biochemical images in three dimensions using positron-emitting radioisotope labels, results in a system capable of ultrasensitive molecular and high-resolution functional imaging, which is invaluable for understanding the physiology and function of the brain.

## Conventional biomedical research

### Basic information

Conventional biomedical research includes the following procedures: observation/result analysis, target discovery, and research for basis/application. In conventional biomedical research, advances in medical diagnostics and treatment depend on a comprehensive understanding of epidemiological findings or pathophysiological processes^40^. Gathering data from studies, evaluations, and interpretations involving humans completes the framework of conventional biomedical research, which has contributed greatly to maintaining human health. In observation/result analysis, researchers use biotechnology to observe biological and pathological differences between disease and normal states^41^, design appropriate experiments to confirm these differences in a controlled system^42^, and subsequently evaluate the experimental results using reliable biotechnology and analyze the results using an appropriate statistical approach^43^. In the target discovery step, the analysis provides targets (specifically, molecular targets)^44^. In the next step, a technology for regulating the discovered targets is developed, the mode of action (basic and necessary data on the target regulatory action)^45,46^ is investigated, and the therapeutic potential for diseases is investigated by expanding the range from the cellular level to the entire organism level^47^. Notably, a human-level clinical trial involves multiple stages in which safety and effectiveness are closely examined^48^. This process alone is a long and complex research stage that requires approximately 10 years^49^. New drugs for several diseases are being developed through conventional biomedical research. A summarized history of these drugs is presented in the Supplementary Information, including Supplementary Table 1. We selected some of the major causes of global mortality, as ranked by the World Health Organization between 2019 and 2020, and the representative diseases that have significantly impacted human history^50^.

Considering the latest accomplishments, conventional biomedical research seems to have advantages and disadvantages. It has provided more accurate and detailed knowledge in specific and detailed research fields^51^. Furthermore, in-depth development of research using animal models has been achieved. However, there are some limitations to the conventional approach. First, in most studies, academic scientists with relatively limited clinical knowledge select animal and human participants for observational analyses; therefore, the selection criteria and results remain suboptimal^52^. Furthermore, there may be bias in classifying and comparing healthy participants and those with specific diseases in medical research due to the relative lack of clinical knowledge. Selection bias could lead to shortfalls in knowledge acquisition. Second, interpreting the effects of various drug candidates in preclinical disease animal models for reasons such as the low genetic concordance rate with humans is difficult. The results of preclinical animal studies are often insufficient to directly predict or alleviate human diseases^53,54^. However, prioritizing preclinical animal models is still a reasonable consideration, and several researchers depend heavily on animal models. Third, until recently, most results have been based on short-term cellular and animal experiments; however, these findings likely differ from findings in humans due to various factors. Limitations exist in predicting effectiveness and toxicity in humans because of deficiencies in experimental design strategies and biased species variance; however, animal studies are still performed. Fourth, existing biomedical studies evaluating hypotheses in clinical trials have been conducted on several patients. These hypothesis-driven studies have limitations in investigating heterogeneous and multifactorial diseases, and actual human clinical samples are difficult to collect. Therefore, in unbiased large-scale collection and clinical data analysis, it is challenging to identify patterns and generate actionable predictions regarding disease progression. Moreover, even though the new drug candidates developed through conventional biomedical research undergo numerous expensive tests, only <10% of the compounds have been approved with sufficient efficacy and adequate toxicity results to meet the predictive value of preclinical studies^52^. Table 1 summarizes the advantages and disadvantages of conventional biomedical research.

**Table 1:** Advantages and disadvantages of conventional biomedical research.

Advantages	- Ethical and legal research
	- More accurate and detailed knowledge in specific and detailed research fields
	- In-depth advances in biomedical research using animal models
Disadvantages	- Academic scientists select the participants for observational analysis
	- Animal models (nonhuman level) having a low genetic concordance rate with human models are primarily used in drug development
	- Short-term experiments are the major basis for understanding
	- Clinical trials have limitations in terms of investigating heterogeneous and multifactorial diseases

### Conventional biomedical research on the brain

#### Neurodegenerative diseases

Using conventional biomedical research, the observation and exploration of clinical symptoms led to the discovery of several neurodegenerative brain diseases, such as Alzheimer’s disease (AD), Parkinson’s disease (PD), multiple sclerosis^55–57^, and various systemic diseases. These brain diseases have unique characteristics that can be used to distinguish them from other diseases^58–60^. For example, in 1892, Paul Blocq and Georges Marinesco discovered senile plaques for the first time in the brain of a patient who died from epilepsy^58^. Furthermore, senile plaques were observed in patients with dementia, and in 1910, they were named “Alzheimer’s disease” by a physician named Alois Alzheimer, who observed them along with significant shrinkage of the hippocampus and neurofibrillary tangle^59^. With advancements in technology, the abnormal accumulation of amyloid plaques and tau proteins has become known as a pathological hallmark of AD. Therefore, numerous studies have focused on developing amyloid- and tau-related treatments. However, most of these treatments have failed in clinical trials because the exact causes of amyloid and tau accumulation are still unknown^61^. For AD, 76% of agents in phase III trials in 2016 were disease-modifying therapies, including amyloid- and tau-targeted agents, whereas by 2022, only 29% of the agents in phase III trials were disease-modifying therapies^62,63^. Additionally, AD is reportedly associated with mitochondrial dysfunction, oxidative stress, and neuroinflammation^64,65^, and many studies on the development of treatments for these aspects have been conducted, but the success rate is still extremely low.

Treatments for neurodegenerative diseases such as AD, which have poorly understood underlying mechanisms, are difficult to develop using a conventional biomedical research approach. Therefore, the current treatment for neurodegenerative diseases relies only on clinically observed pathological symptoms and is aimed merely at symptom improvement. For instance, the widespread loss of cholinergic neurons and overactivation of *N*-methyl-D-aspartate (NMDA) receptors in the brains of patients with AD have been identified, and cholinesterase inhibitors and NMDA receptor antagonists are commonly used to alleviate AD symptoms^66^. In patients with PD, the loss of dopaminergic neurons in the midbrain, dopamine precursors, dopamine agonists, and L-3,4-dihydroxyphenylalanine decarboxylase inhibitors have been identified, and catechol-O-methyl transferase (COMT) inhibitors are used to relieve PD symptoms^67^. Current treatments for neurodegenerative diseases provide symptomatic relief, but there is no conclusive evidence that they can fundamentally cure the disease. Additionally, because neurodegenerative diseases are gradually progressive, patients are on medication for prolonged durations, leading to the need for increased dosages and various side effects^68^. Therefore, novel treatments that differ from the currently used conventional methods should be developed. Table 2 summarizes the clinically approved drugs for treating neurodegenerative diseases^69–73^.

**Table 2:** Clinically approved drugs for treating neurodegenerative diseases.

Conditions	Therapeutic drugs	Mode of action	Reference
Alzheimer’s disease	Donepezil	Cholinesterase inhibitor	^69,70^
	Rivastigmine		
	Galantamine		
	Memantine	NMDA receptor antagonist	
Parkinson’s disease	Levodopa	Dopamine precursor	^71,72^
	Pramipexole	Dopamine agonist	
	Ropinirole		
	Selegiline	Dopamine agonist MAO-B inhibitor	
	Rasagiline		
	Entacapone	COMT inhibitor	
Multiple sclerosis	Teriflunomide	Dihydro-orotate dehydrogenase inhibitor	^73^
	Dimethyl fumarate	Protects against oxidative stress-induced cellular injury and loss	
	Fingolimod	Sphingosine 1-phosphate receptor agonist	

*COMT* catechol-O-methyl transferase, *MAO-B* monoamine oxidase B, *NMDA* *N*-methyl-D-aspartate.

#### Psychiatric disorders

Mental illnesses have been described since ancient times; however, any true understanding of their nature was impossible, as they were considered a supernatural phenomenon caused by displeased gods, eclipses, curses, or sin^71^. As with most brain diseases, experience-based treatments and exploration of the molecular mechanisms involved in psychiatric disorders occurred almost simultaneously.

Psychiatric disorders began to be established from a somatogenic perspective in the 19th century^74^. Chlorpromazine (CPZ) was originally synthesized as a possible potentiator of general anesthesia and was accidentally developed as an antipsychotic drug^75^. Henri Laborit, a French surgeon, used cocktail lytique to prevent surgical shock and observed that when 50–100 mg of CPZ was injected intravenously, it prevented shock and induced relaxation and sedation without loss of consciousness^76^. Since then, CPZ has been significantly effective in relieving hallucinations, delusions, and disorganized thought in patients with schizophrenia^77^. Since the development of CPZ, many drugs, including thioridazine, haloperidol, and pimozide, with similar effects have been synthesized, but their molecular mechanism was unknown. Then, it was discovered that these drugs bind to dopamine receptors^78^. Therefore, because clomipramine, a tricyclic antidepressant, was found to be effective in reducing obsessive symptoms, the serotonin hypothesis for obsessive-compulsive disorder was proposed^79,80^. A role of gamma-aminobutyric acid (GABA) in mood disorders was proposed based on the clinical observation that valproic acid was effective in patients with bipolar disorders^81^. Psychiatric disorders are estimated to account for 13% of the global burden of disease, surpassing cardiovascular diseases and cancer^82^. However, accurate mechanisms have not yet been discovered, and only a few patients receive basic treatment^83^. Translational research can reveal novel treatments; however, it requires a coordinated effort at the disciplinary and national levels.

#### Other brain diseases

Attention deficit hyperactivity disorder (ADHD) is a neurodevelopmental disorder that affects 5% of children globally^84^. Its exact cause is unknown, but many risk factors, including norepinephrine and dopamine imbalances, have been identified^85^. The most common and effective medications to regulate norepinephrine are psychostimulants, including methylphenidate and dexamphetamine^85,86^. These treatments are effective; however, they have many side effects, such as decreased appetite, behavioral rebound, irritability, sleep problems, and tic exacerbation^87,88^. Epilepsy is one of the most common neurological disorders, affecting approximately 65 million people globally^89,90^. The exact cause of epilepsy is unknown; however, it is closely associated with neurodegeneration, ADHD, and stroke^91–93^. There have been significant advances in epilepsy treatment, including calcium ion channel and GABA transporter modulators, over the past few decades; however, one-third of patients are still fighting the disease, even with the currently available medications^94^. As described above, there are different types of brain diseases, including neurodegenerative, psychiatric, and neurodevelopmental disorders, but their treatments overlap and are limited. This is because the exact etiologies are unknown, and in most cases, the cause has been identified as an imbalance of neurotransmitters, including dopamine and serotonin. Therefore, a paradigm shift is needed in translational brain research. Brain diseases are more complex than systemic diseases. Consequently, special attention should be given to interdisciplinary efforts that provide a comprehensive view of the entire brain. Table 3 summarizes the available drugs for psychiatric disorders and other brain diseases, excluding treatments that are also used for various psychiatric diseases^84,95–103^.

**Table 3:** Clinically approved drugs for psychiatric and other brain diseases.

Conditions	Therapeutic drugs	Mode of action	Reference
Schizophrenia	Chlorpromazine	D_2_ receptor antagonist	^95,96^
	Haloperidol		
	Risperidone		
	Olanzapine		
Obsessive-compulsive disorder	Clomipramine	Serotonin reuptake inhibitor	^97,98^
	Fluvoxamine	CYP2C19 inhibitor (inhibits the metabolism of clomipramine)	
	Citalopram	Serotonin reuptake inhibitor	
	Fluoxetine	Selective serotonin reuptake inhibitor	
Bipolar disorder	Lithium carbonate	Dopamine neurotransmission inhibitor	^99,100^
	Valproic acid	Voltage-gated sodium channel blocker	
	Carbamazepine	Sodium channel blocker	
ADHD	Methylphenidate	Dopamine and noradrenaline transporter inhibitor	^84,101^
	Dexamphetamine	Dopamine reuptake inhibitor	
Epilepsy	Phenobarbitone	Barbiturate	^102,103^
	Carbamazepine	Sodium channel blocker	
	Diazepam	GABA receptor agonist	

## Emerging technologies

### Neuroimaging

Neuroimaging is a noninvasive technique that is used to scan brain structures or functions in humans and animals at the macro level. There are various promising tools for brain imaging: MRI, functional MRI (fMRI), PET, electroencephalography (EEG), and magnetoencephalography. The simultaneous provision of different types of important information, such as structural, functional, and molecular information and temporal changes, makes neuroimaging an emerging high-throughput analysis toolkit.

MRI uses nuclear magnetic resonance to create images of brain structures^39^, whereas fMRI uses blood oxygenation level-dependent contrast to observe the degree and area of brain activation in humans^10^. PET observes changes in metabolic processes and blood flow by measuring positrons emitted by radioactive tracers^11^. EEG measures the brain’s electrical activity mainly generated by nerve cells^104^, whereas magnetoencephalography measures the magnetic field change derived from the brain’s electrical activity^105^.

In the history of neuroimaging, blood flow changes have been associated with brain function^106^. This has underpinned the significant progress in functional brain imaging with the development of fMRI and PET over the past 30 years^107^. When the brain structures are damaged, brain function can be disrupted. This is because the neural system is substantially flexible and organizes neural networks through regional interactions. Conversely, it is locally rigid and maintains the specificity of neural responses to brain functions that are specialized to separate regions. These properties underlie the principles describing the brain’s functional organization: segregation and integration^108^. Functional segregation was defined as “localizationism” by Franz-Joseph Gall (1758–1828), Johann Spurzheim (1776–1832), and Paul Pierre Broca (1824–1880), who stated that a function is specialized to a particular anatomical region. Therefore, injuries or lesions in that region can cause loss of function.

Conversely, according to Marie Jean Pierre Flourens (1794–1867), Kurt Goldstein (1878–1965), and Karl Lashley (1890–1958), functional integration implies networks of interactions among specialized regions. The loss of intact connections in a network causes functional loss. Deficient global integration or local segregation is associated with functional brain organization. Therefore, the functional manifestations of local brain regions have been increasingly used to understand brain diseases. Consequently, recent neuroimaging methods can be used as a translational approach in neuroscience to investigate how brain structures and functions are linked to genetic variations and disease manifestations.

Combining basic neuroscience, neuroimaging, and clinical applications to develop diagnostic methods for brain diseases has recently emerged as an intermediary approach in neuroscience. Therefore, a better understanding of the symptoms of brain disease is becoming possible^109^. Notably, numerous neuroimaging studies have attempted to diagnose neurovegetative diseases such as AD, PD, amyotrophic lateral sclerosis, and chronic traumatic encephalopathy. Recent advances in neuroimaging techniques and data analysis methods that provide the means to test the underlying organization of brain structure (MRI), function (fMRI), and metabolism (PET) from the microscopic to the macroscopic level have enabled this research. Therefore, brain injuries and neurodegenerative brain diseases can be linked through translational neuroimaging, which may provide new insights for biomedical research. For example, traumatic brain injury (TBI) studies using animal models are becoming increasingly important to match neuroimaging findings in humans with pathophysiological results in animals^110^. The idiosyncratic features of human TBI, such as heterogeneity, severity, temporal pathophysiology, and different brain systems, challenge the clinical application of mild TBI in animal models^111^. Nevertheless, translational neuroimaging results may explain the similarities and differences between humans and animals in terms of the effects of TBI^112^. In particular, resting-state fMRI in humans and mice demonstrated dynamic functional changes in mild TBI, wherein deficits and recovery occur over time^112,113^. Therefore, neuroimaging plays an important role in translational neuroscience because it is the cornerstone of in vivo measurements. However, its use for the collection and utilization of other types of data is limited.

The high cost of using neuroimaging methods such as MRI and PET can make translational neuroscience based on neuroimaging difficult; therefore, using a large database led by neuroimaging consortia, such as the UK Biobank (*n* = 35,735), the Human Connectome Project (*n* = 1200), and the Alzheimer’s Disease Neuroimaging Initiative (*n* > 1800), is increasingly essential^114^. The more neuroimaging data that are shared, the more studies can progress by sharing thousands of individuals’ MRI, fMRI, and PET data for basic neuroscience, neuroimaging, and clinical studies. A large amount of data has been used to derive reliable results in replicability and longitudinal studies^115,116^.

Therefore, recent neuroimaging studies have attempted to combine multimodal^117–120^, and multispecies^121,122^ data to interpret brain structural and functional changes based on the underlying mechanism^123^. The advantages of multimodal neuroimaging include high spatiotemporal resolution, improved data quality, and understanding of the anatomical basis of functional activity. However, the disadvantages are different resolutions, data complexities, and sample sizes^123–125^. Nevertheless, whether neuroimaging results are consistent with the results derived from different data types remains unclear due to the current lack of mining of multimodal data.

### Multiomics

Technological advances in high-throughput platforms for omics-based analysis, including genomics, transcriptomics, proteomics, metabolomics, and lipidomics, have greatly contributed to understanding human health, medicine, and diseases^126^. Genomics and transcriptomics identify genetic variants and multifactorial targets associated with diseases; however, predicting the biological effects of individual variants is difficult due to epigenetic, transcriptional, and posttranslational modifications. Proteomics quantifies protein abundances and posttranslational modifications such as glycosylation, phosphorylation, and ubiquitination^127^. With improved mass spectrometry-based methods, thousands of proteins in a patient’s tissues or body fluids can be identified simultaneously. Therefore, proteomics can provide comprehensive information about actual protein functions and cellular processes associated with disease pathogenesis. However, compared with genomics, proteomics still has insufficient coverage at the genome level due to several technical issues (such as ionization efficiency for poorly responding peptides)^128^. Moreover, there are weak correlations between each type of omics data (transcripts versus proteins), mostly reflecting reactive processes, such as cellular half-life, RNA/protein degradation, splicing, and posttranslational modifications^129^. Since the etiology of most diseases involves multiple factors, it is impossible to focus on one factor, and diagnosis and treatment are difficult^128^. Therefore, comprehensive technology integration is required to identify disease-related factors. The integrative approach combines individual omics data sequentially or simultaneously to understand molecular/intermolecular interactions^130^. Metabolomics, especially lipidomics, uses mass spectrometric techniques similar to those of proteomics but analyses the products of metabolism, in which enormous numbers of metabolites vary with disease state^131–136^.

To date, multiomics approaches have been applied to cancer biology. In cancer diagnosis and treatment alone, multilevel information, such as mutation, fusion gene, RNA, and protein level expression changes, is needed^137,138^. Omics analyses have helped to elucidate key mechanisms involved in cancer onset, treatment resistance, and the risk for recurrence. Notably, integrative multiomics analyses have provided more comprehensive molecular signatures to identify cancer subtypes^139,140^. Recent advances in omics analysis and data archiving and processing have provided reliable data^141^. There have been global efforts to obtain new molecular information by which to treat and diagnose cancers by reproducibly obtaining and integrating omics information^142^. The Cancer Genome Atlas (TCGA) of the United States National Cancer Institute (NCI) provides multiomics datasets (including genomic, transcriptomic, epigenomic, proteomic, and phosphoproteomic data) from >20,000 patients across 33 cancer types to aid in the discovery of molecular signatures to diagnose, treat, and prevent cancer^143^. The International Cancer Genome Consortium (ICGC), a genomics and informatics consortium that started in 2007, began the 25 K project for genome analysis of 25,000 primary untreated cancers^144^. Analysis of whole-genome cancer (Pancancer Analysis of Whole Genomes project) started in 2013, and > 3000 eligible whole-cancer genomes of several cancer types are currently being analyzed^144,145^. The ICGC aims to accelerate genomic oncology research (Accelerating Research in Genomic Oncology project), where key clinical queries and patient clinical data drive the inquisition of cancer genomes. Clinical trials provide a unique resource of multiomics data from patients with cancer to accelerate the discovery of new therapies^146^. The Clinical Proteomic Tumor Analysis Consortium was formed and centered on the U.S. NCI by applying proteomics technology^147^. Tumor Analysis Consortium databases contain all the clinical information of patients with cancer^147,148^. Notably, all proteomics-based technologies, such as sample preparation, peptide generation, chemical labeling, mass spectrometry, and data processing, are optimized and shared^149^. The Tumor Analysis Consortium project has created and provided novel proteomic results for cancer biomarkers and targets^150,151^. Projects to discover new mechanisms and target molecules through multiomics analysis of diverse diseases are being actively undertaken as joint research between countries^151^.

Analysis of trace molecules in single cells is becoming the aim for identifying causes of diseases. Due to efforts such as the development of single-cell isolation methods and the improvement of cell resolution, the omics layer can be analyzed integrally at the single-cell level^152,153^. Additionally, multiomics technology development has greatly increased our understanding of the critical pathways that influence complex cell physiology and secondary metabolite production^154^. Multiomics involves comparing and interpreting vast amounts of experimental data and performing customized statistical analysis; therefore, it is time-consuming, requires professional manpower, and imposes a considerable overall economic burden. However, the diversification of analysis, interpretation, and visualization of multiomics data to overcome these limitations has led to improvements in methods, devices, and processes^130,155^.

Multiomics also involves several computational techniques for the integrative analysis of multiomics datasets. An unsupervised model-based method, multiomics factor analysis, integrates multiple datasets and finds principal sources of variability^156^. iCluster, capable of identifying genomic features that mostly influence biological variation, uses joint latent variable models to characterize molecular subtypes^157^. It also successfully synthesizes the complexity of multiomics data through machine learning (ML), deep learning (DL), and network-based feature extraction and transformation method development^158,159^. Multiomics advances are revolutionary; therefore, incorporating truly integrated multiomics analysis can rapidly advance precision medicine. Efforts are ongoing to develop an analytical infrastructure to effectively create, analyze, and annotate multiomics data^160^.

### Artificial intelligence

The data obtained from basic and clinical research for translational studies are heterogeneous and large in scale^161,162^. These data span from the microscale to the macroscale, include human and animal data, and include molecular, cellular, regional, whole-brain, behavioral, and even textual information^163,164^. Therefore, innovative methods may be required to integrate and process these complex data.

Notably, various artificial intelligence (AI) types have been suggested since John McCarthy coined the term “artificial intelligence” in 1956^165,166^. In particular, ML, which enables learning from experience, has various applications, including classification, prediction, and generalization through supervised or unsupervised routes^167^. In the medical field, it has been successfully applied to determine the prognosis and diagnosis of diseases^166,168^. However, the performance of conventional ML significantly depends on the feature selection and extraction process, which DL does not require^169^. Recently, DL, a type of ML, has grown exponentially, supported by the development of various algorithms, big data, and hardware such as graphic processing units^170–172^. DL involves a kind of neural network with multiple and deep layers; however, it can learn from raw data, features on hidden layers, and results^173^.

Thus far, DL has successfully performed neuroscience analysis, including analyses of DNA/RNA sequences, metabolomic data, proteomic data, microscopy images, and MRI data^174–180^. For example, DL has been successfully applied to drug discovery, which is a costly and time-consuming process. DL can identify drug targets, biomarkers, and druggability^181^. Moreover, DL can be used to confirm drug-target interactions and drug‒drug combinations^182,183^. DL is a high-throughput tool because the large chemical and protein space makes it difficult for conventional methods to search for and identify the characteristics of any appreciable fraction of all possible combinations^182,183^. Generative DL techniques have recently emerged and have been applied in neuroscience. Since the generative adversarial network was introduced in 2014^184^, many generative adversarial network variants have been developed to classify diseases and for disease progression modeling and synthetic data generation^185^. Generative adversarial networks can produce plausible data. Therefore, a small amount of data for EEG, MRI, and multimodal neuroimages were augmented^186–188^, and missing multiomics data could be handled by generative DL^189,190^.

**Fig. 1:**
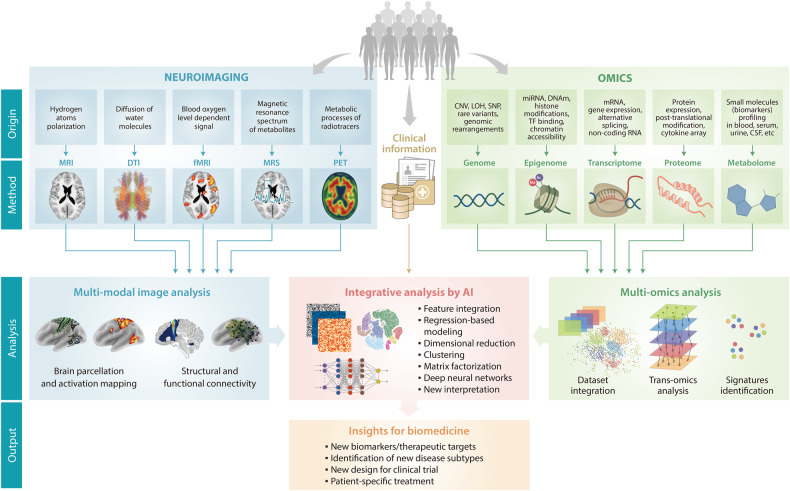
Workflow for new biomedical research using high-throughput analysis tools, including neuroimaging, multiomics, and AI technology. Neuroimaging, including MRI, DTI, MRS, and PET, provides multimodal imaging information. Multiomics, including genomics, proteomics, and metabolomics, provides molecular information. By linking with clinical information, AI-based integrative data analysis using multimodal neuroimaging and multiomics offers valuable new insights into biomedical brain research, such as new biomarkers, disease subtypes, and treatments. MRI magnetic resonance imaging, AI artificial intelligence, PET positron emission tomography, DTI diffusion tensor imaging, MRS magnetic resonance spectroscopy, SNP single-nucleotide polymorphism, CNV copy number variation, LOH loss of heterozygosity, fMRI functional MRI.

The next step for translational research involves developing methods to handle heterogeneous and multimodal data, a problem of algorithms, hardware, and computational systems. Nevertheless, we expect progress to be rapid and on a large scale with promising outcomes. Emerging high-throughput analysis tools, including neuroimaging, multiomics, and AI, can help researchers conduct large-scale biomedical research that induces a paradigm shift for conventional research. Figure 1 presents a streamlined workflow for new biomedical research using high-throughput analysis tools.

## New directions in translational research

With conventional research methods that proceed from basic to clinical, we can fully understand living organisms; however, these methods have limitations in overcoming diseases^191^. Furthermore, academic fields have been self-focused and fragmented due to relatively slow technological advances in scientific fields, including intercommunication methods^141^. However, academic research fields are maturing and achieving success in applications based on robust fundamental backgrounds, such as electronic engineering. Based on this maturity of academic research fields and intercommunication tools, the current era is truly of smart communication.

A new era is coming for biomedical research, representing a paradigm shift^192^. We have presented emerging high-throughput analysis tools such as neuroimaging, multiomics and AI technology^193–195^. These tools enable patient-centered and large-scale biomedical research^196^ and can ultimately drive a new approach. In new translational research, various high-throughput analyses of patients, clinical data utilization, personalized diagnostics, and treatment through the discovery of target molecules by multiomics are involved. Furthermore, the emergence of interdisciplinary cooperative studies involving basic science and engineering will provide insight into how unresolved questions in biomedicine can be solved^197,198^. Interdisciplinary collaborative studies could include core capability enhancement by securing large-scale data, development of analysis technology, and application of AI technology to data integration and interpretation.

**Fig. 2:**
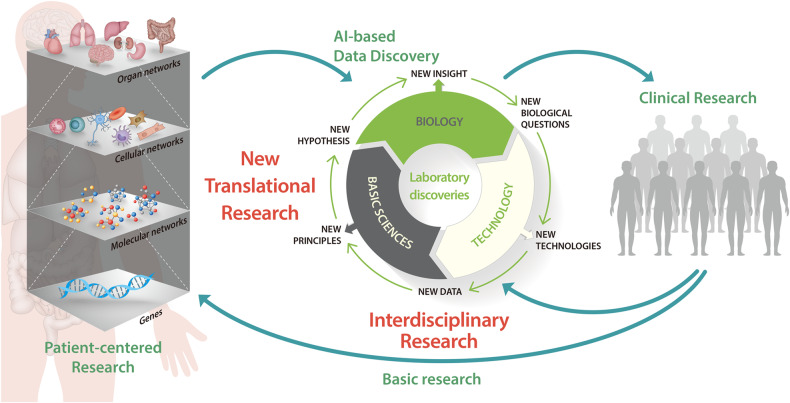
Scheme of the paradigm shift required for translational research combined with interdisciplinary research. New directions in translational research start with multilayered patient-centered research using high-throughput analysis tools such as multiomics and imaging with high reproducibility and reliability. AI-based data integration and interpretation reveal new molecular and signaling signatures. Subsequently, the discovered information is inputted into the interdisciplinary collaborative research cycle. The relevant conclusions from an interdisciplinary collaboration provide new technologies for diagnoses and treatments. Then, new technologies are applied to clinical research using patient groups. The obtained information is fed back to the cycle to improve the conclusions. AI artificial intelligence.

Figure 2 presents a scheme of new translational research combining interdisciplinary collaborative research. It starts with multilayered patient-centered research and includes patient data acquisition steps on micro- and macroscales^163,164^. Since this step requires multilayered analysis with high reproducibility and reliability, high-throughput analysis tools such as multiomics and imaging and corresponding clinical data should be employed^141^. In this step, multilayered diverse information determines the next step: interdisciplinary collaborative research. AI-based data integration and interpretation are used to discover molecular and signaling signatures before the next step^143^. Subsequently, the discovered information is put into the interdisciplinary collaborative research cycle (Fig. 2), which includes interdisciplinary research fields such as biology, basic sciences, and technology. Each research field takes the discovered information from AI-based analysis and draws its interpretation from its point of view. Excluding biology, each of these interpretations would be unique because multilayered and patient-centered data constitute a new data type and a previously unknown principle in each research field. These findings will provide new hypotheses and insights into diseases. Therefore, subsequent experimental approaches to validate new hypotheses are needed. This step would reveal new technology questions, thereby involving the technology field. The technology field has developed new technologies and provides new data for functional research against the new hypotheses. In this cycle, each research field communicates with the others to draw more rational and advanced conclusions in their field and harmoniously across diverse disciplines. This activity promotes the theoretical advancement of each discipline and new approaches, theories, and technologies. The new technologies for diagnostics and treatments derived from this step are then applied to clinical research. This step is not a conventional clinical trial but rather the stage for testing new technologies using a group of patients with no ethical concerns, similar to the test bed scale^198^. From the clinical research step, valuable information is acquired from human specimens^199^. The acquired information is fed back to the interdisciplinary collaborative research cycle to improve their conclusions. The information is also fed back to the multilayered patient-centered research step to help improve analytical methods and availability determination.

As an example, when PD is considered in this scheme, the first step can consist of the data analysis of patients with PD (Supplementary Fig. 1). To acquire molecular information, proteogenomics, metabolomics, and transcriptomics of biological sources, including cerebrospinal fluid, blood, and urine, are applied^200–202^. Neuroimaging tools such as MRI, PET, or single-photon emission computed tomography are used to obtain macroscale information^116^. Subsequently, the corresponding clinical information of patients with PD is collected. After the fundamental analysis for data validation, this multilayered information is put into an AI-based in-depth analysis for big data integration. This step discovers well-suited and/or new target molecule and signal module candidates. Next, the results are compared and verified with previously well-known information, such as dopamine pathway-related contents^143^. In interdisciplinary collaborative research, the integrated information, including images and molecular and signal signatures, is used as new data for each research field. New hypotheses and insights from each research field will appear using the data, and preliminary interdisciplinary research on new PD targets will be conducted. The development of technology for PD diagnosis and treatment will also commence. New molecular and/or imaging targets will be discovered; therefore, the technology field can provide previously unknown diagnostic and treatment methods for PD, such as noninvasive diagnosis technology and target molecule regulation-based treatment using deep brain stimulation technology^203,204^. Members of the interdisciplinary cycle could correct their hypotheses and develop technologies through active intercommunication and discussion. Next, these valuable outcomes are applied to clinical research. The new findings and technologies associated with PD will be applied to limited human samples and interpreted: the technologies for precise PD diagnostics/treatment and noninvasive deep brain stimulation-based PD treatment can be tested. After analyzing the clinical research results, a new research design for in-depth basic and interdisciplinary research will be devised and executed. The acquired information is fed back to the interdisciplinary collaborative research cycle to revise the PD hypothesis and calibrate preliminary technologies, including diagnostics and deep brain stimulation.

Recent psychiatric studies have identified the necessity and feasibility of the new translational research that we suggest here. Notably, all multiomics, neuroimaging, and AI methods were not integrated; however, there were some uses of partial integration for clinical neuroscience. For example, multimodal neuroimaging (MRI and PET) and machine learning have been integrated to predict psychiatric disorders and neurodegenerative diseases^205–207^. PET and DL (convolutional neural network) have been integrated to differentiate patients with AD^208^. Another study reported the integration of EEG and machine learning algorithms to detect predementia AD^209^. In other studies, combining multimodal neuroimaging and multiomics was used to link neuroimaging markers with biomarkers of neurodegeneration, indicating a greater genetic risk for AD. For example, neuroimaging markers in patients with AD correlated with neurodegeneration biomarkers, such as GFAP and Ptau 181 and 217^210^. Diverse combinations of neuroimaging and multiomics have been used to classify patients with PD using DL^211^ and to predict patients with PD using network analysis-based proteomics^212^. Furthermore, some studies have integrated transcriptomic and neuroimaging brain models^213^ and neuroimaging-based connectomics to predict neurodegenerative processes^214^. These attempts may bridge the gap between genomics and neuroimaging and find biomarkers for treating neurodegenerative diseases^193^. These studies align with the new direction we suggest, including interdisciplinary research concepts of biology; however, in basic sciences and technology, partial integration may not address several limitations derived from conventional brain research. Therefore, considering brain structures, functions, and genes across individuals through the lens of integrated multiomics, neuroimaging, and AI information may be increasingly crucial for understanding the underlying mechanisms of brain diseases, including neurodegenerative and psychiatric disorders, and for developing treatments^215^. Thus, new translational research may be able to solve difficult problems in brain biomedical research.

In conclusion, conventional biomedical research has made numerous contributions^198^. However, its limitations are apparent, especially in biomedical brain research. To date, theories and technologies have rapidly developed and matured. Therefore, a new direction in translational research combined with the application of new technologies and interdisciplinary collaborative research will inevitably overcome the limitations of conventional approaches for brain research.

### Supplementary information

Supplementary Information
