# Supplementary material for: Paradigm shift required for translational research on the brain
Source: Exp Mol Med. 2024 May 1;56(5):1043–54. doi: 10.1038/s12276-024-01218-x (PMC11148129; doi:10.1038/s12276-024-01218-x)
Supplement: Supplementary file 1 — Supplementary Information [file 12276_2024_1218_MOESM1_ESM.pdf]

Supplementary Information

Application of conventional biomedical research

Ischemic heart disease, which is the most common cause of global mortality, occurs when the heart does not receive sufficient blood supply due to coronary artery blockage. Risk factors of coronary artery blockage include high blood pressure, diabetes, and obesity. Symptoms such as chest tightness, shortness of breath, and indigestion may occur. Drug treatment for ischemic heart disease is administered to reduce oxygen consumption by increasing the blood flow and decreasing the heart rate via coronary artery dilation. Aspirin and prasugrel are also administered to prevent blood clotting, whereas beta blockers, calcium-channel blockers, and nitrates are provided to lower the blood pressure. Cilnidipine is a promising fourth-generation dual L/N-type calcium-channel blocker and a dihydropyridine calcium antagonist. This product was jointly developed by Fuji Viscera Pharmaceutical Company and Ajinomoto and was approved for marketing in 1995. Cilnidipine at molecular concentrations effectively inhibits N-type calcium-channel currents in isolated sympathetic neurons.[1]

Cancer is a disease of uncontrolled cell division. While the cause of cancer is not simply a single factor, it occurs because the cell cycle is not regulated due to mutations in the genes of normal cells or cancer suppressor genes. Cancer can occur in all organs, including the colon, stomach, liver, lungs, prostate, and breasts, with lung cancer having the highest incidence according to the 2014–2018 statistics by the National Cancer Institute (NCI) of the United States.[2] Lung cancer is classified into non-small cell carcinoma, squamous cell carcinoma, adenocarcinoma, large cell carcinoma, and small cell carcinoma according to the microscopic morphology of the cancer cells. With respect to treatment, it is classified into small cell lung cancer (SCLC) and non-small cell lung cancer (NSCLC). Chemotherapy is widely used as treatment for NSCLC, and many targeted therapies have been developed. Crizotinib is available using the anaplastic lymphoma kinase (ALK) inhibitor principle, which has been applied to actively develop new drugs in recent years. Approximately 5% of NSCLCs exhibit *ALK* gene rearrangement.[3,4] Such *ALK* gene rearrangement produces abnormal ALK proteins

that allow cells to irregularly grow and spread. ALK inhibitors target these abnormal ALK proteins. Crizotinib is an inhibitor of receptor tyrosine kinases, including ALK, that prevents *ALK* gene rearrangement.

Diabetes mellitus is a metabolic disease caused by an insulin secretion abnormality and is mainly characterized by hyperglycemia. Its symptoms include thirst, increased urine output, and weight loss. Diabetes mellitus can be managed with insulin treatment (type-1 diabetes mellitus) or lifestyle modification and drug treatment (type-2 diabetes mellitus). Among hypoglycemic agents for regulating insulin secretion, sodium–glucose co-transporter 2 (SGLT2) inhibitors are recently developed drugs that suppress glucose re-absorption in the kidneys.[5] Dapagliflozin is a representative new drug that was developed by Bristol Myers Squibb in collaboration with AstraZeneca and approved in the United States in 2014.[6] Dapagliflozin inhibits sub-type 2 of sodium–glucose transport protein, which control more than 90% of glucose re-absorption in the kidney. Blocking this transport mechanism removes blood glucose through the urine.[7]

With respect to diarrheal diseases, common symptoms include loose watery stools, dehydration, lower abdominal pain, and cramping. Factors expressing viruses (rotavirus, Norwalk virus, or hepatitis virus) and bacteria (*Shigella* species and *Escherichia coli*) have been found based on these symptoms, and drug targets have been researched and developed. For instance, the diarrheal disease caused by microsporidia is cured by albendazole.[8]

Albendazole, invented by Robert J. Gyuric and Vassilios J. Theodorides in 1975, is used to treat various parasitic diseases such as hookworm, threadworm, and roundworm infections.[9] The mechanism of action (MOA) is based on specific binding to tubulin, which results in the destruction of the microtubule function and interferes with the microtubule-mediated transport of secretory vesicles in helminths.[10] Although it is sufficiently effective to be listed as an essential drug by the World Health Organization, the number of cases developing resistance to repellents has considerably increased in various countries such as Brazil and Uruguay.[11]

Tuberculosis (TB) is the deadliest infectious disease in human history and is a chronic infection

caused by *Mycobacterium tuberculosis* complex. The symptoms of TB are similar to those of colds and lung diseases, making it difficult to diagnose in several cases, and these differ depending on the site of infection; however, respiratory symptoms (e.g., cough, phlegm, and chest pain) usually appear, whereas non-specific symptoms (e.g., fever, irritability, and appetite loss) may appear. Streptomycin was evaluated for TB using the standard logic method in modern randomized controlled trials. Streptomycin originates from *Streptomyces griseus* and was first isolated in 1943 by Albert Schatz; since then, it has been widely considered as a first-line treatment for TB.[12] Streptomycin interferes with the binding of the 30S subunit of formyl-methionyl-tRNA and binds to the bacterial ribosome S16 protein, thereby inhibiting protein synthesis and leading to the death of microbial cells.[13] Thiacetazone, which was developed based on mycobacteria cultured from patients exhibiting recurrence,[14] has been used for treatment since the 1960s. Thiacetazone acts as a bacteriostatic agent by inhibiting the synthesis of mycolic acid cyclopropane and prevents the bacteria in TB from spreading by interfering with cell wall functions. While thiacetazone is rarely used alone as treatment because of toxicity issues, it is used to prevent resistance to stronger drugs and is still administered in several African and South American countries because of its low cost.[15]

Patients with malaria exhibit signs of acute febrile infection, such as fever, chills, nausea, abdominal pain, and shortness of breath. The most common and deadly causative species of malaria is *Plasmodium falciparum*.[16] Artemisinin (ATC) was discovered by Tu Youyou in 1972 to combat malaria caused by *P. falciparum*. Currently, complication-free ATC-based combination therapy has been adopted as the standard of care for malaria. ATC interacts with digested hemoglobin molecules to form free radicals, thereby killing the parasite. Recent published results have revealed that ATC interacts with PfATP6 in protozoan cells to prevent cellular death due to increased calcium concentration.[17] More effective new drugs in combination with lumefantrine as the most common partner drug have been developed. Coartem manufactured by Novartis is a binder of artemether (an ATC derivative) and lumefantrine. Coartem has been reported to inhibit the synthesis of nucleic acids and proteins via suppression of β-hematin formation.[18] It suppresses the pathogens' resistance and shows a better effect than ATC.[19] Additionally, studies have presented examples of combinations with

various partner drugs.[20]

As there are various types of influenza, its symptoms, treatment methods, and target drugs are also diverse. Representative examples of drugs include rimantadine for influenza A viruses and baloxavir marboxil for influenza A and B viruses.[21] Currently, the most recognized antiviral drugs, such as oseltamivir, simultaneously act on both influenza A and B viruses.[22] Discovered in 1963 and patented by William W. Prichard, rimantadine binds to an amino acid of the M2 transmembrane channel and blocks proton transport, thereby suppressing influenza activity and viral replication.[23] In 2018, baloxavir marboxil was manufactured with a new MOA by Shionogi Co., Ltd. (a Japanese pharmaceutical company) and Roche AG (a company based in Switzerland). With respect to its MOA, baloxavir marboxil works by inhibiting viral replication by inactivating the function of an endonuclease encoded by the PA subunit of the viral polymerase complex.[21] Oseltamivir (brand name: Tamiflu) was developed by Gilead Sciences, Inc., with shikimic acid as a starting material and was approved for medical use in 1999 based on the results of a randomized double-blind trial.[24] Regarding its MOA, oseltamivir interferes with the release of influenza particles from infected cells by binding to neuraminidase, resulting in an abated spread of influenza viruses in the respiratory tract.[25]

A cure for acquired immunodeficiency syndrome (AIDS) is being continuously sought. Weight loss, chronic diarrhea, and non-specific symptoms may appear in infected individuals or they may be asymptomatic following human immunodeficiency virus (HIV) infection. In asymptomatic cases and those showing non-specific symptoms, the presence of HIV infection is difficult to determine, and there exists a high possibility of infecting others. There are various causes of HIV/AIDS. Among them, zidovudine, an antiretroviral drug, is used for prevention and treatment of identified causes. Uniquely, zidovudine induces hematological toxicity.[26] Lamivudine/zidovudine was approved by the Food and Drug Administration in 1997 as the first fixed-dose combination antiviral marketed under the brand name Combivir.[27] The side effects of using zidovudine alone have been dramatically improved.[28] With respect to the MOA, lamivudine and zidovudine in Combivir competitively reduce the reverse transcriptase activity, resulting in reduced number of viruses in infected HIV cells.[29]

Supplementary Table 1. Application of conventional biomedical research for diseases

Diseases	Therapeutic drugs	Mode of action	Reference
**Ischemic heart disease**	Cilnidipine	Inhibits N-type calcium-channel currents in isolated sympathetic neurons at ultrafine molecular concentrations	1
**Cancer (lung)**	Crizotinib	Inhibits receptor tyrosine kinases, including ALK, thereby preventing the rearrangement involved in lung cancer cell survival	3
**Diabetes mellitus**	Dapagliflozin	Inhibits sub-type 2 of the sodium-glucose transport protein, which regulates glucose re-absorption in the kidney.	7
**Diarrheal diseases**	Albendazole	Disrupts the microtubule function and interferes with the microtubule-mediated transport of secretory vesicles in helminths	10
**Tuberculosis**	Streptomycin	Interferes with the binding of the 30S sub-unit of formyl-methionyl-tRNA and binds to the bacterial ribosome S16 protein	13
	Thiacetazone	Inhibits the synthesis of mycolic acid cyclopropane	15
**Malaria**	Artemisinin	Interacts with PfATP6 in protozoan cells to prevent cellular death due to increased calcium concentration	17
	Lumefantrine	Inhibits the synthesis of nucleic acids and proteins via suppression of β-hematin formation	18
**Influenza**	Rimantadine	Suppressed influenza activity and viral replication result from binding to the amino acids of the M2 transmembrane channel and blocking proton transport	23
	Baloxavir	Inhibits viral replication by inactivating	21

	marboxil	the function of an endonuclease encoded by the PA sub-unit of the viral polymerase complex.	
	Oseltamivir	Interferes with the release of influenza particles from infected cells by binding to neuraminidase, resulting in an abated spread of influenza viruses in the respiratory tract	25
**HIV/AIDS**	Lamivudine/zidovudine	Inhibit the reverse transcriptase activity, causing HIV-infected cells to decrease the number of viruses	29

ALK, anaplastic lymphoma kinase; HIV, human immunodeficiency virus; AIDS, acquired immunodeficiency syndrome

Supplementary Fig. 1. Application of new translational research strategy on Parkinson's disease (PD). In step 1, patients with PD are analyzed using a neuroimaging tool and omics technology. In step 2, an artificial intelligence (AI) -based in-depth data analysis for integration and interpretation provides new PD signatures. In step 3, a new hypothesis for diagnosing and treating PD is derived through interdisciplinary collaborative research along with the development of technologies for diagnosis and treatment. In step 4, new technologies discovered in the previous step are clinically applied to test the bed scale of patients with PD. After the clinical research, in steps 5 and 6, in-depth basic and interdisciplinary research will be designed through a feedback process. It helps revise PD treatment and improve ease of use.

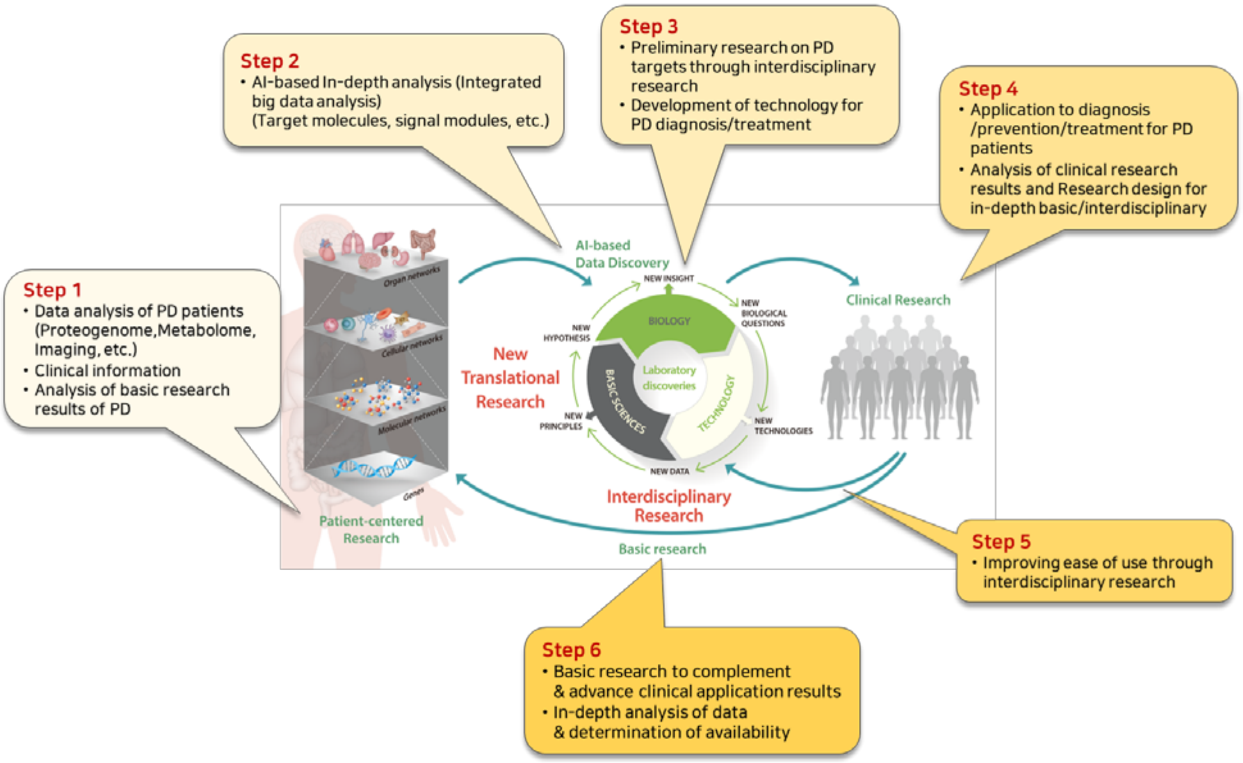

References

1 Chandra, K. S. & Ramesh, G. The fourth-generation Calcium channel blocker: cilnidipine. *Indian Heart J.* **65**, 691-695 (2013).

2 seer.cacer.gov. Annual Report to the Nation 2021: Overall Cancer Statistics. (National Cancer Institute, 2021).

3 Sahu, A., Prabhash, K., Noronha, V., Joshi, A. & Desai, S. Crizotinib: A comprehensive review. *South. Asian. J. Cancer.* **2**, 91-97 (2013).

4 Miyamoto, S. *et al.* Transformation to small-cell lung cancer as a mechanism of acquired resistance to crizotinib and alectinib. *Jpn. J. Clin. Oncol.* **46**, 170-173 (2016).

5 Komoroski, B. *et al.* Dapagliflozin, a novel SGLT2 inhibitor, induces dose-dependent glucosuria in healthy subjects. *Clin. Pharmacol. Ther.* **85**, 520-526 (2009).

6 Anderson, S. L. Dapagliflozin efficacy and safety: a perspective review. *Ther. Adv. Drug. Saf.* **5**, 242-254 (2014).

7 Dandona, P. Minimizing Glycemic Fluctuations in Patients with Type 2 Diabetes: Approaches and Importance. *Diabetes Technol. Ther.* **19**, 498-506 (2017).

8 Azam, A., Peerzada, M. N. & Ahmad, K. Parasitic diarrheal disease: drug development and targets. *Front. Microbiol.* **6**, 1183 (2015).

9 Ainsworth, S. B. *Neonatal Formulary: Drug use in Pregnancy and the First Year of Life*. seventh edn, Vol. 64 (John Wiley & Sons, 2014).

10 Králová, V. *et al.* Antiproliferative effect of benzimidazole anthelmintics albendazole, ricobendazole, and flubendazole in intestinal cancer cell lines. *Anticancer. Drugs.* **24**, 911-919 (2013).

11 Entrocasso, C. *et al.* Clinical efficacy assessment of the albendazole–ivermectin combination in lambs parasitized with resistant nematodes. *Vet Parasitol* **155**, 249-256 (2008).

12 Comroe Jr, J. H. Pay dirt: the story of streptomycin: Part I. From Waksman to Waksman. *Am. Rev. Respir. Dis.* **117**, 773-781 (1978).

13 Sharma, D., Cukras, A. R., Rogers, E. J., Southworth, D. R. & Green, R. Mutational analysis

of S12 protein and implications for the accuracy of decoding by the ribosome. *J. Mol. Biol.* **374**, 1065-1076 (2007).

14 Keshavjee, S. & Farmer, P. E. Tuberculosis, drug resistance, and the history of modern medicine. *N. Engl. J. Med.* **367**, 931-936 (2012).

15 Alahari, A. *et al.* Thiacetazone, an antitubercular drug that inhibits cyclopropanation of cell wall mycolic acids in mycobacteria. *PLoS. One.* **2**, e1343 (2007).

16 Antinori, S., Galimberti, L., Milazzo, L. & Corbellino, M. Biology of human malaria plasmodia including Plasmodium knowlesi. *Mediterr. J. Hematol. Infect. Dis.* **4**, e2012013 (2012).

17 Eckstein-Ludwig, U. *et al.* Artemisinins target the SERCA of Plasmodium falciparum. *Nature* **424**, 957-961 (2003).

18 Combrinck, J. M. *et al.* Insights into the role of heme in the mechanism of action of antimalarials. *ACS. Chem. Biol.* **8**, 133-137 (2013).

19 Byakika-Kibwika, P., Lamorde, M., Mayanja-Kizza, H., Khoo, S. & Merry, C. Artemether-lumefantrine combination therapy for treatment of uncomplicated malaria: the potential for complex interactions with antiretroviral drugs in HIV-infected individuals. *Malar. Res. Treat.* **2011**, 703730 (2011).

20 Biamonte, M. A., Wanner, J. & Le Roch, K. G. Recent advances in malaria drug discovery. *Bioorg. Med. Chem. Lett.* **23**, 2829-2843 (2013).

21 O'Hanlon, R. & Shaw, M. L. Baloxavir marboxil: the new influenza drug on the market. *Curr. Opin. Virol.* **35**, 14-18 (2019).

22 Hata, A., Akashi-Ueda, R., Takamatsu, K. & Matsumura, T. Safety and efficacy of peramivir for influenza treatment. *Drug. Des. Devel. Ther.* **8**, 2017-2038 (2014).

23 Vorobjev, Y. N. An effective molecular blocker of ion channel of M2 protein as anti-influenza a drug. *J. Biomol. Struct. Dyn.* **39**, 2352-2363 (2021).

24 Jefferson, T. *et al.* Neuraminidase inhibitors for preventing and treating influenza in adults and children. *Cochrane. Database. Syst. Rev.* **2014**, CD008965 (2014).

25 Moscona, A. Neuraminidase inhibitors for influenza. *N. Engl. J. Med.* **353**, 1363-1373 (2005).

26 Muluken, W. & Epherem, M. Assessment of the prevalence of zidovudine induced anemia among adult HIV/AIDS patients on HAART in an Ethiopian Hospital. *Occup. Med. Health. Aff.* **6**, 2 (2018).

27 World Health Organization, G., Switzerland. (WHO model formulary 2008 2009).

28 Portsmouth, S. D. & Scott, C. J. The renaissance of fixed dose combinations: Combivir. *Ther. Clin. Risk. Manag.* **3**, 579 (2007).

29 Anderson, P. L. & Rower, J. E. Zidovudine and lamivudine for HIV infection. *Clin. Med. Rev. Ther.* **2**, a2004 (2010).
